# Decline in the Recovery from Synaptic Depression in Heavier *Aplysia* Results from Decreased Serotonin-Induced Novel PKC Activation

**DOI:** 10.1371/journal.pone.0136907

**Published:** 2015-08-28

**Authors:** Tyler William Dunn, Wayne S. Sossin

**Affiliations:** Department of Neurology and Neurosurgery, Montreal Neurological Institute, McGill University, Montreal, Quebec H3A 2B4, Canada; Texas A&M University—Corpus Christi, UNITED STATES

## Abstract

The defensive withdrawal reflexes of *Aplysia* are important behaviors for protecting the animal from predation. Habituation and dishabituation allow for experience-dependent tuning of these reflexes and the mechanisms underlying these forms of behavioral plasticity involve changes in transmitter release from the sensory to motor neuron synapses through homosynaptic depression and the serotonin-mediated recovery from depression, respectively. Interestingly, dishabituation is reduced in older animals with no corresponding change in habituation. Here we show that the cultured sensory neurons of heavier animals (greater than 120g) that form synaptic connections with motor neurons have both reduced recovery from depression and reduced novel PKC Apl II activation with 5HT. The decrease in the recovery from depression correlated better with the size of the animal than the age of the animal. Much of this change in PKC activation and synaptic facilitation following depression can be rescued by direct activation of PKC Apl II with phorbol dibutyrate, suggesting a change in the signal transduction pathway upstream of PKC Apl II activation in the sensory neurons of larger animals.

## Introduction

Habituation and dishabituation of the defensive withdrawal reflexes of the marine mollusk *Aplysia* have long been examined as simple modifiable behaviors arising from simple underlying neurophysiological changes. Electrophysiological recordings in semi-intact preparations determined that a significant portion of the change in behavior can be attributed to changes in synaptic efficacy of the sensory to motor neuron synapses involved in the reflex [1,2]. The dramatic reduction in postsynaptic potential (PSP) amplitude with low frequency stimulation and the recovery from that depression with either nerve shock or serotonin (5-hydroxytryptamine, 5HT), are the result of presynaptic changes in transmitter release [3,4,5]. The synaptic depression and the subsequent recovery from depression can also be observed at synapses formed between presynaptic sensory neurons and postsynaptic motor neurons in culture using low frequency stimulation of the sensory neuron and exogenous 5HT [6]. While the mechanisms of these two forms of plasticity have yet to be fully elucidated, it is known that behavioral dishabituation requires PKC activity in the sensory neuron [7]. More specifically, recovery from synaptic depression was shown to depend on 5HT activation of the novel PKC Apl II [8].

Indiscriminate use of animals for isolating neurons used for synaptic co-cultures results in widely variable measures of 5HT facilitation following synaptic depression. Since older/heavier animals were shown to have reduced dishabituation [9,10], we considered the possibility that some of the observed variability in the recovery from depression could arise from an age or size dependent change in the animal. Indeed, we found that sensory neurons isolated from animals >120g failed to show full recovery from synaptic depression with 5HT, in agreement with behavioral data showing reduced dishabituation in these animals [9]. We also observed less 5HT mediated translocation of expressed eGFP-tagged PKC Apl II to the membrane in sensory neurons isolated from the heavier animals indicating a change at or upstream of PKC activation. Most importantly, direct PKC activation with the phorbol ester PDBu largely rescued the translocation of eGFP-PKC Apl II and the recovery from synaptic depression in sensory neurons isolated from larger animals. The data overall suggests that the reduction in dishabituation in heavier/ older animals is related to a change in the signal transduction pathway that activates PKC downstream of 5HT in presynaptic sensory neurons.

## Materials and Methods

### Animals and Cell Culture

All animals were obtained from the NIH/University of Miami National Resource for *Aplysia* mariculture facility. Our holding tanks are approximately 300 gallons, held at 15°C, with weekly 10% volume replacement and salinity measurements. Tank pH is kept between 7.8 and 8.2 with sodium bicarbonate.

Animals were weighed prior to use, anesthetized with isotonic MgCl_2_ and ganglia removed. Ganglia were digested for approximately two hours in Dispase II (Roche) at 34°C with specific times and enzyme concentration adjusted between lots of enzyme, for quality of neuron isolation. Abdominal ganglia tended to have digestions times ten minutes less than pleural-pedal ganglia. All sensory neurons used here were pleural ganglia ventral caudal cluster sensory neurons and motor neurons were abdominal ganglia LFS siphon motor neurons. Synaptic co-cultures were positioned with overlapping axons in dishes alone or such that only one sensory neuron contacts one motor neuron. Neurons were cultured for at least 72hr in 50% modified L-15 media (added salts for *Aplysia*) and 50% hemolymph, supplemented with glutamine. Nuclear injections of eGFP-PKC Apl II in pNEX3 vectors into sensory neurons were done 18–24hr before use; the injected cultured neurons were placed in the fridge at 4°C four hours after injection, and removed 1hr before use as previously described [11,12]. All other cultures were kept at room temperature in containers >60% humidity until use.

### Electrophysiology

Sharp electrode, current-clamp voltage recordings of presynaptic and postsynaptic neuron membrane potentials were made at isolated synaptic pairs or isolated sensory neurons in culture as described previously in [13]. Sharp glass electrodes 15–25MΩ backfilled with 2M potassium acetate, were manipulated to the cells with Sutter MP-225 micromanipulators (Sutter Instruments) and membrane potentials amplified with an Axoclamp 900A with signals digitized using a Digidata 1440A and pClamp software (Molecular Devices). The culture media was replaced with an *Aplysia* recording saline of the following composition in (mM): NaCl (460), MgCl_2_ (55), CaCl_2_ (10), KCl (10), D-Glucose (10), HEPES (10), pH 7.6. LFS motor neurons were first impaled and membrane potentials held at -80mV following confirmation of LFS identity (see [14]), rebalancing of the electrode, and injection of hyperpolarizing pulses to monitor input resistance. Pleural sensory neurons were always impaled second as initial entry with a 2ms buzz can sometimes initiate an action potential which would generate the very important first postsynaptic potential (PSP) PSP1. This first PSP can still be measured provided the buzz duration is sufficiently short and the motor neuron was impaled first. Presynaptic sensory neurons were also hyperpolarized to -80mV between stimuli. LFS motor neuron PSPs were evoked by generating action potentials in the pleural sensory neurons at a frequency of 0.05Hz with 50ms depolarizing pulses.

### Fluorescence imaging

Confocal imaging of eGFP-PKC Apl II localization was done using a Zeiss LSM-510, running Zen software (Carl Zeiss Inc). Before the experiment, eGFP-PKC Apl II expression was confirmed in the cell of interest with epifluorescence and a standard FITC filter set. Then, a confocal optical section through the center of the sensory neuron soma was taken before and after addition of either 10**μ**M 5HT and/ or 200nM phorbol dibutyrate (PDBu) with laser and PMT settings maintained constant throughout the trial. Pixel dwell time and averaging were adjusted to get the best quality image in approximately 30s.

### Data analysis

PSP amplitude or rise-rates were measured using pClamp software. If PSPs activated postsynaptic voltage-activated currents (usually when greater than 20mV), initial PSP rise-rates were measured [15]. PSP amplitude or rise-rate was normalized as a percentage of the first PSP (%PSP1) in each trial. eGFP-PKC Apl II translocation was measured as the difference in fluorescence at the membrane (F_mem_) and the fluorescence in the cytosol (F_cyto_) using ImageJ to make measurements [11,16]. All data values are mean values ± standard error of the mean. Statistical analyses were preformed with Graph Pad Prism.

## Results

### 5HT-mediated recovery from homosynaptic depression is reduced with sensory neurons isolated from animals >120g without changes in 5HT-mediated increases in sensory neuron excitability

**Fig 1:**
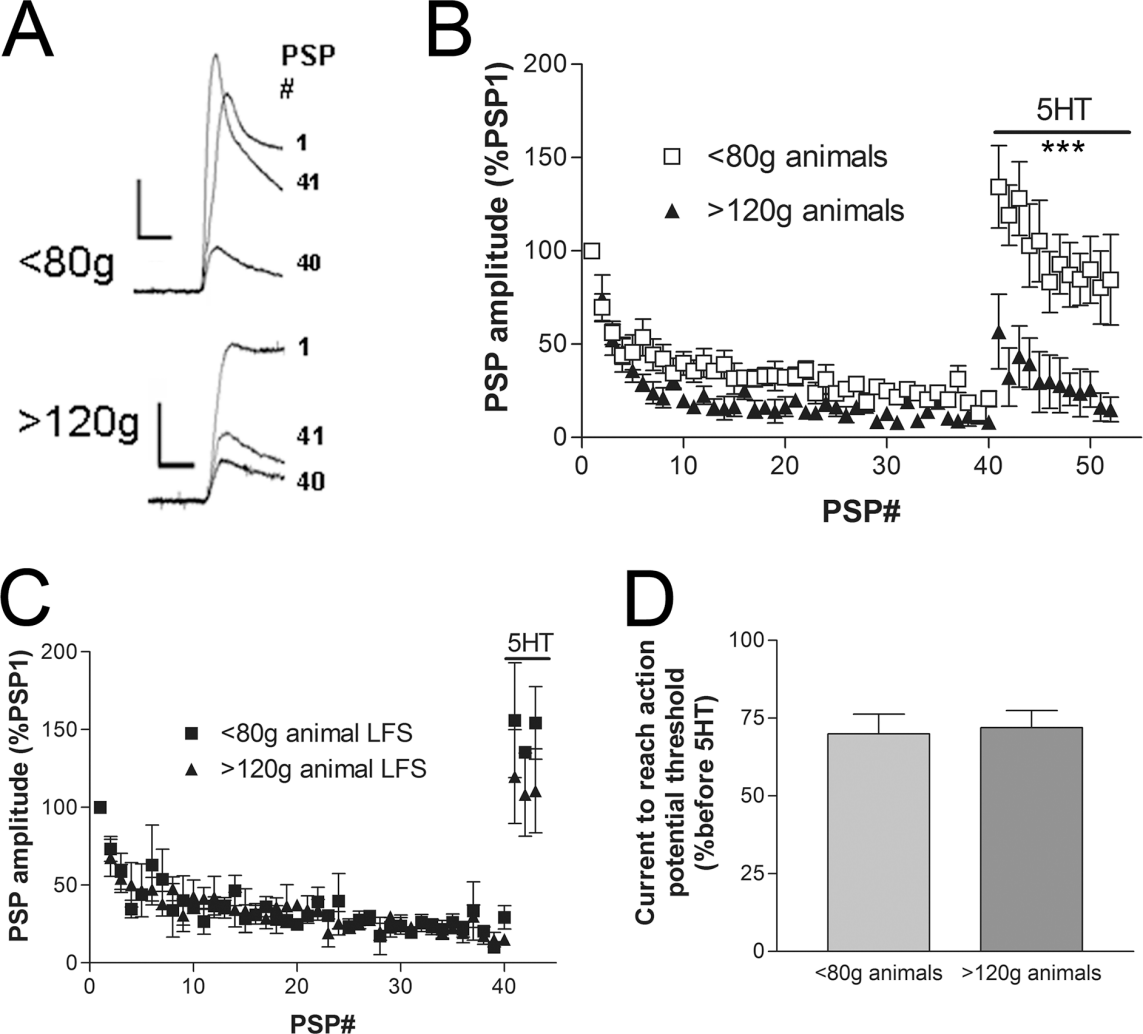
Reduction in 5HT-mediated recovery from synaptic depression with sensory neurons from animals larger than 120g. (A) Voltage traces of sensory neuron action potential evoked PSPs recorded in the motor neuron at synaptic connections with the sensory neuron isolated from an animal less than 80g (top traces) and greater than 120g (bottom traces). Only traces of PSP1 (first PSP), PSP40 (40th PSP, depressed PSP), and PSP41 (41st PSP, recovery from depression with 5HT) are presented. Scale bars are 10/8mV and 10ms. (B) Time course of the change in PSP amplitude with 0.05Hz stimulation, resulting in depression, and subsequent recovery from depression with 5HT after PSP40. Use of sensory neurons from animals greater than 120g results in significantly less facilitation/recovery from depression with 5HT (Comparing the two data sets with a two-way ANOVA and Bonferroni posttests, PSP1-PSP40 P>0.05 and PSP41-PSP52 P<0.001 at all PSPs except PSP46 at P<0.01, n = 5 synaptic connections with small animal sensory neurons and n = 7 synaptic connections with sensory neurons from large animals). (C) Using sensory neurons from animals <80g, varying the size of the animal from which the postsynaptic motor neurons were isolated had no effect on the recovery from synaptic depression with 5HT. Data are from three synapses in each group. (D) The amount of depolarizing current injected into the sensory neuron to produce an action potential is reduced following the addition of 5HT (presented as the amount of current required to fire the action potential to produce PSP41 as a percentage of the current required to fire the action potential to produce PSP40). The reduction in the stimulating current in the sensory neuron was equally observed with both sensory neurons of <80g and >120g animals.

Homosynaptic depression of *Aplysia* sensory to motor neuron synapses occurs with low frequency stimulation (0.05Hz) of single action potentials in the sensory neuron, and observed as a dramatic reduction in motor neuron PSP amplitude (Fig 1A and 1B). While examining the reversal of homosynaptic depression with 5HT, we noticed a trend as synapses made from larger animals tended to show incomplete recovery from synaptic depression with 5HT. To directly test this possibility we compared synaptic connections formed with sensory neurons from animals <80g with sensory neurons isolated from animals >120g. When <80g animals were used to isolate sensory neurons, addition of 5HT to synapses previously depressed led to the complete recovery of synaptic depression back to the initial synaptic strength (Fig 1A and 1B). However, when sensory neurons were isolated from animals >120g, the recovery from depression with 5HT was significantly reduced (Fig 1A and 1B). Such a reduction in facilitation following depression was not observed when the postsynaptic motor neuron was isolated from animals >120g and paired with sensory neurons from smaller animals, <80g (Fig 1C). This is consistent with the presynaptic locus of the mechanisms underlying both homosynaptic depression and the 5HT-mediated recovery from depression [3,4,5].

The initial PSP amplitude varies widely at synaptic connections reformed between *Aplysia* sensory and motor neurons in culture. However, the initial PSP amplitudes with sensory neurons from <80g animals were not significantly different from the synapses formed with sensory neurons from animals >120g (30.6±7.4mV and 20.1±5.0mV compared with a t-test, P>0.05). Furthermore, the initial synaptic strength was not significantly correlated to the amount of depression observed (Pearson r = -0.5912 for <80g, and Pearson r = 0.1069 for >120g) or the amount of facilitation with 5HT (Pearson r = -0.7302 and Pearson r = 0.7185 for >120g) (all P>0.05).

Activation of the 5HT receptor positively coupled to adenylate cyclase results in an increase in excitability of the sensory neuron that is independent of the mechanism underlying the recovery from synaptic depression [6,17]. This increase in excitability can be observed partly as a reduction in the amount of current required to reach action potential threshold in the sensory neuron. In the same trials used to examine the recovery from synaptic depression in Fig 1B, the reduction in the amount of current required to fire an action potential in the sensory neuron with 5HT was similarly observed at the sensory neurons from both the small and large animals (Fig 1D).

**Fig 2:**
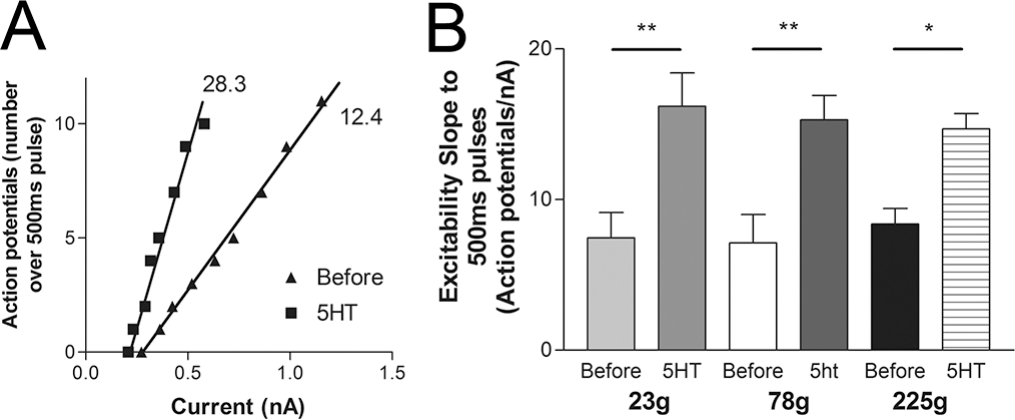
Increase in membrane excitability with 5HT in three different sized animals. (A) Counts of the number of action potentials during 500ms depolarizing pulses of varying current amplitudes in a representative pleural sensory neuron from an animal of approximately 100g. The slope of this relationship is a more sensitive indicator of changes in membrane excitability than measuring action potential threshold alone. The increase in excitability of the sensory neuron with 5HT can be measured from the change in the slope. (B) Sensory neurons isolated from an animal of three distinct sizes, small 78g, a larger animal 225g and a very small animal at 23g. A significant increase in excitability measured with the number of action potentials per nA of depolarizing current was observed at sensory neurons from all three animals (Comparing before and 5HT with a two-way ANOVA with Bonferroni post tests at all three animal weights, *P<0.05, **P<0.01). Data from sensory neurons from single animals in each group (n = 6 sensory neurons examined from each animal). A one-way ANOVA of the initial slopes (before) found no significant difference between the three animals.

As a variety of channels affecting membrane excitability are modulated by 5HT, a more sensitive measure of the change in membrane excitability is to measure the change in the number of action potentials observed with 500ms depolarizing pulses ([13]; Fig 2A). The change in threshold is measured from the x-intercept and can be a small change compared to the change in the slope of the number of action potentials observed per nanoamp of depolarizing current, which is more sensitive to change (Fig 2A). Isolated sensory neurons from either a 23g, 78g, or 225g animal had similar initial excitability slopes and were similarly excited by 5HT, confirming that 5HT changes in membrane excitability are not affected by the size of the animal from which the sensory neurons were isolated (Fig 2B).

### The degree of recovery from synaptic depression correlates with the size of the animal from which the sensory neurons were isolated

**Fig 3:**
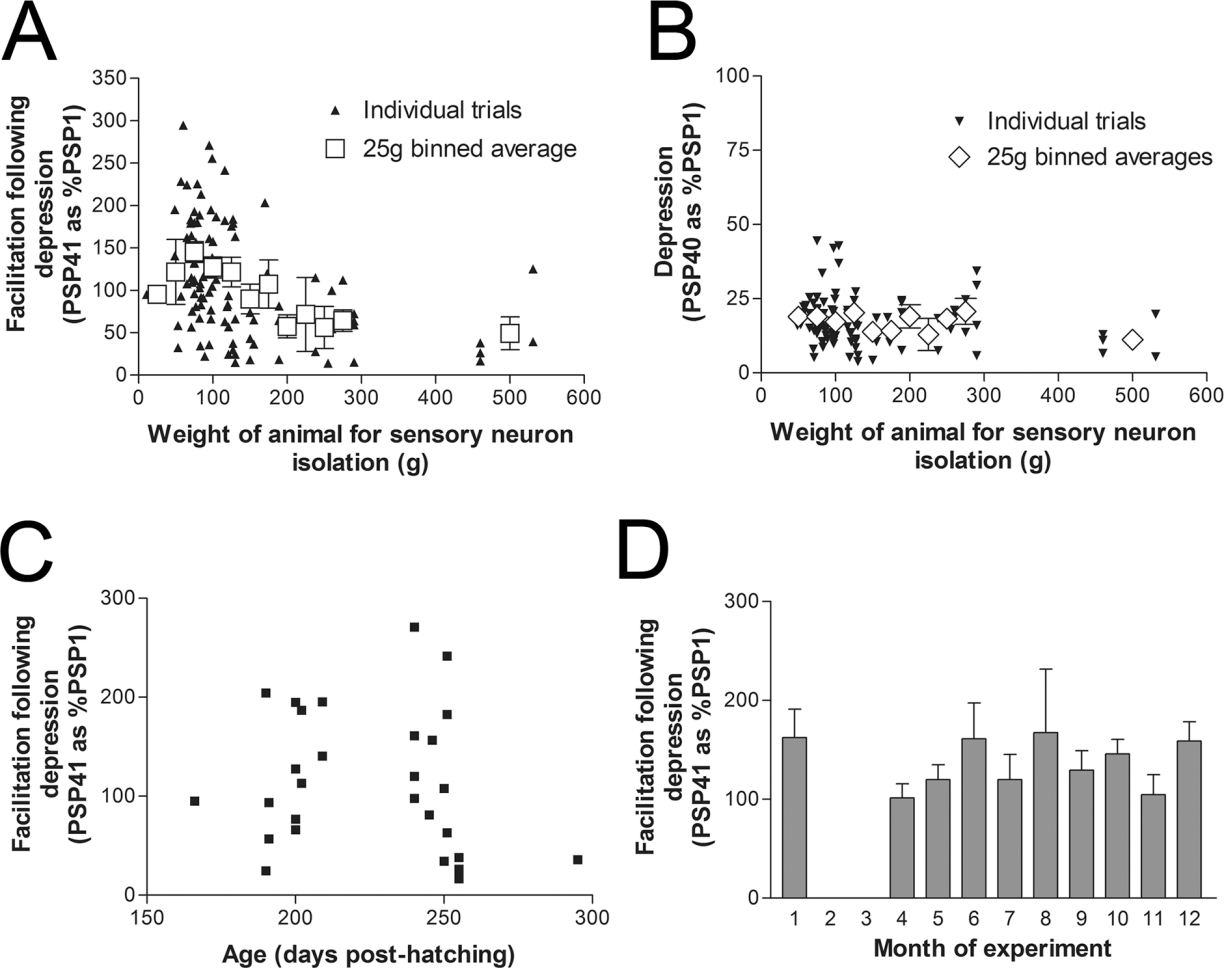
Correlation between the weight of the animal and synaptic depression or subsequent facilitation with 5HT. (A) The amount of facilitation following homosynaptic depression (PSP41 as a percentage of PSP1) was significantly correlated with the weight of the animal the sensory neurons were isolated from (Pearson r = -0.4087, 107 synapses with results averaged for each animal, n = 47 animals, P<0.01). (B) The amount of depression (the amplitude of PSP40 as a percentage of PSP1) did not correlate with the weight of the animal (Pearson r = -0.1973, n = 47, P = 0.19). Results from the individual recordings plotted as black triangles (but averaged for each animal for statistical analysis) and grey boxes are 25g binned averages in both A&B. (C) For thirty of the synapses recorded in A and B, the age of the animals from hatching dates are known. When the amount of facilitation with 5HT following synaptic depression is compared with the post-hatching age of the animal the sensory neurons were isolated from there is no correlation (Pearson r -0.2943, n = 12 animals, P = 0.33). (D) Bar graph of the amount of recovery from synaptic depression with 5HT grouped according to the month the experiment was conducted. There is no significant difference in the recovery from depression and the month of the experiment as measured with a one-way ANOVA, P = 0.1, same data set presented in A&B.

When examining a large data set, the weight of the animal used for sensory neuron isolation strongly correlated with the amount of facilitation following depression observed with 5HT (Fig 3A, Pearson r = -0.4087, n = 47, P<0.005) but not with the amount of depression observed with low frequency stimulation (Fig 3B, Pearson r = -0.1973, n = 47, P = 0.19). This data set represents over a hundred synaptic connections with sensory neurons from 47 different animals used at different times over the course of ten consecutive months. We know the specific hatching dates and therefore the exact age of the animals used for the isolation of sensory neurons for thirty of the synapses recorded and presented in Fig 3A and 3B. These sensory neurons were isolated from 12 different animals from 6 different cohorts (egg masses). In examining this portion of the data set, we find no correlation between animal post hatching age and the amount of facilitation observed following synaptic depression (Fig 3C, Pearson r -0.2943, P = 0.33). It is important to note that animal weight and post hatching age was not well correlated in this data set (Pearson r = 0.3112, n = 12 animals, P = 0.32), mostly due to variable lengths of time in planktonic and juvenile stages, and different growth rates (see discussion). Most of the animals were approximately 100g (10 animals averaging 93±6g, one animal 11g, and one animal of 460g), so with only one large animal in the group, animal size did not correlate with facilitation in this data subset (Pearson r = -0.2255, P = 0.48). While the bulk of the data in this subset distribute over two months, a relatively long duration for adult animals, most of the animals are small and therefore do not show the reduction in facilitation. Furthermore, we did not observe a seasonal influence as there was no relationship between the month the experiment was conducted and the amount of facilitation seen with 5HT following depression (Fig 3D).

Many of the 100–200g animals used to generate Fig 3, grew to this weight by spending longer periods of time in our holding tanks. As we are not a mariculture facility, we considered that the change in the observed recovery from depression may be related to the amount of time an animal spends in our holding tanks before use. To address this, three animals were raised in our holding tanks from 20g to between 60–80g before isolating sensory neurons for culture. This increase in animal weight took four to five weeks. Synapses from four sensory neurons were examined, where we found an average depression to 22.0±6.0% of PSP1 at PSP40, consistent with previous measurements of depression (as in Fig 1). After the forty action potential depression, 5HT application produced strong facilitation at these synapses (PSP41 190.4±48.0% of PSP1). This indicates that the length of time an animal spends in our holding tanks is not related to the observed changes in the recovery from depression at synapses formed from sensory neurons of heavier animals, >120g.

### The translocation of eGFP-tagged PKC Apl II with 5HT is reduced in sensory neurons isolated from animals >120g

Facilitation at depressed synapses by 5HT requires activation of an unidentified 5HT receptor coupled to the activation of the novel PKC Apl II [6,8,18]. PKC becomes activated by translocating from the cytoplasm to the plasma membrane. This translocation is due to the binding of PKC to lipids embedded in the membrane, including diacylglycerol, phosphatidic acid and phosphatidylserine for PKC Apl II [11,19]. Thus, we monitored PKC Apl II activation by measuring fluorescent eGFP-tagged PKC Apl II distribution pre and post 5HT treatment as previously described [11,12,18,20].

**Fig 4:**
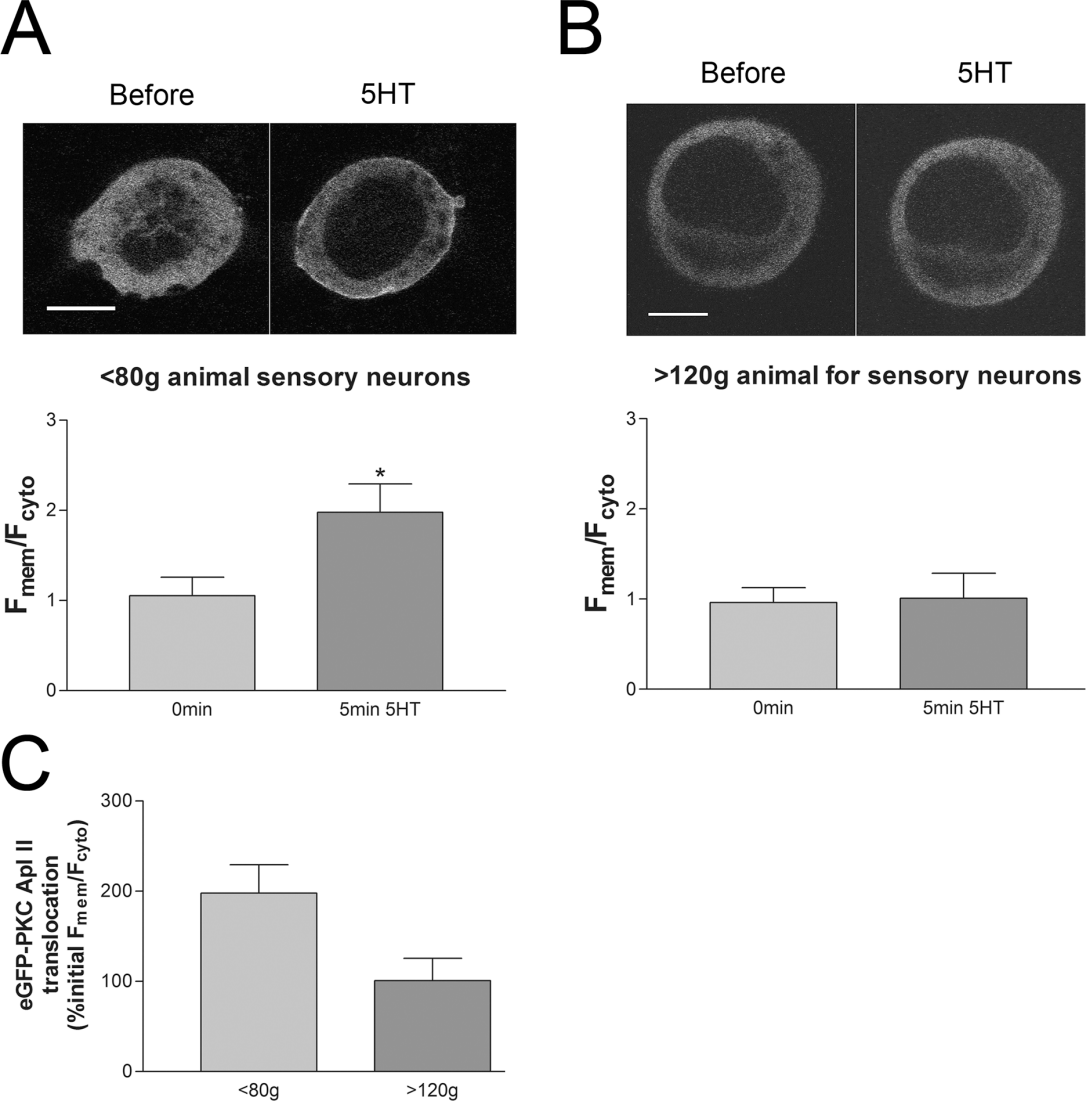
Translocation of eGFP-tagged PKC Apl II with 5HT in sensory neurons from animals <80g or >120g. (A) Representative confocal images of eGFP-PKC Apl II before and after 5min in 10μM 5HT in sensory neuron somata in culture from an animal <80g. The ratio of the eGFP fluorescence intensity at the membrane (F_mem_) to the cytosol (F_cyto_) is greatly increased with 5HT. F_mem_/F_cyto_ was significantly increased with 5HT, P<0.05, (15 sensory neurons from n = 7 different animals). (B) eGFP-PKC Apl II translocation from the cytosol to the plasma membrane was not observed with sensory neurons from animals larger than 120g. The F_mem_/F_cyto_ was not significantly changed with 5HT measured at 5min compared to before 5HT, P>0.05. (11 sensory neurons from n = 5 different animals). Data in AB compared with a two-way ANOVA and Bonferroni post tests. (C) Comparison of the change in eGFP-PKC Apl II translocation with 5HT in sensory neurons from <80g or >120g animals. The F_mem_/F_cyto_ ratio with 5HT is normalized as a percent before 5HT. Scale bars are 20**μ**m.

Sensory neurons expressing eGFP-PKC Apl II were imaged with confocal microscopy before and after addition of 5HT (Fig 4A). The translocation of the kinase to the plasma membrane after 5HT addition was measured as an increase in the membrane-to-cytosol ratio. This increase was significantly smaller in the sensory neurons isolated from animals >120g compared to the sensory neurons isolated from animals <80g (Fig 4B and 4C). These data suggests alteration of the signaling pathways upstream or at the level of PKC activation in the heavier animals.

### Translocation of eGFP-tagged PKC Apl II and recovery from synaptic depression observed in sensory neurons from animals >120g are rescued by the phorbol ester PDBu

**Fig 5:**
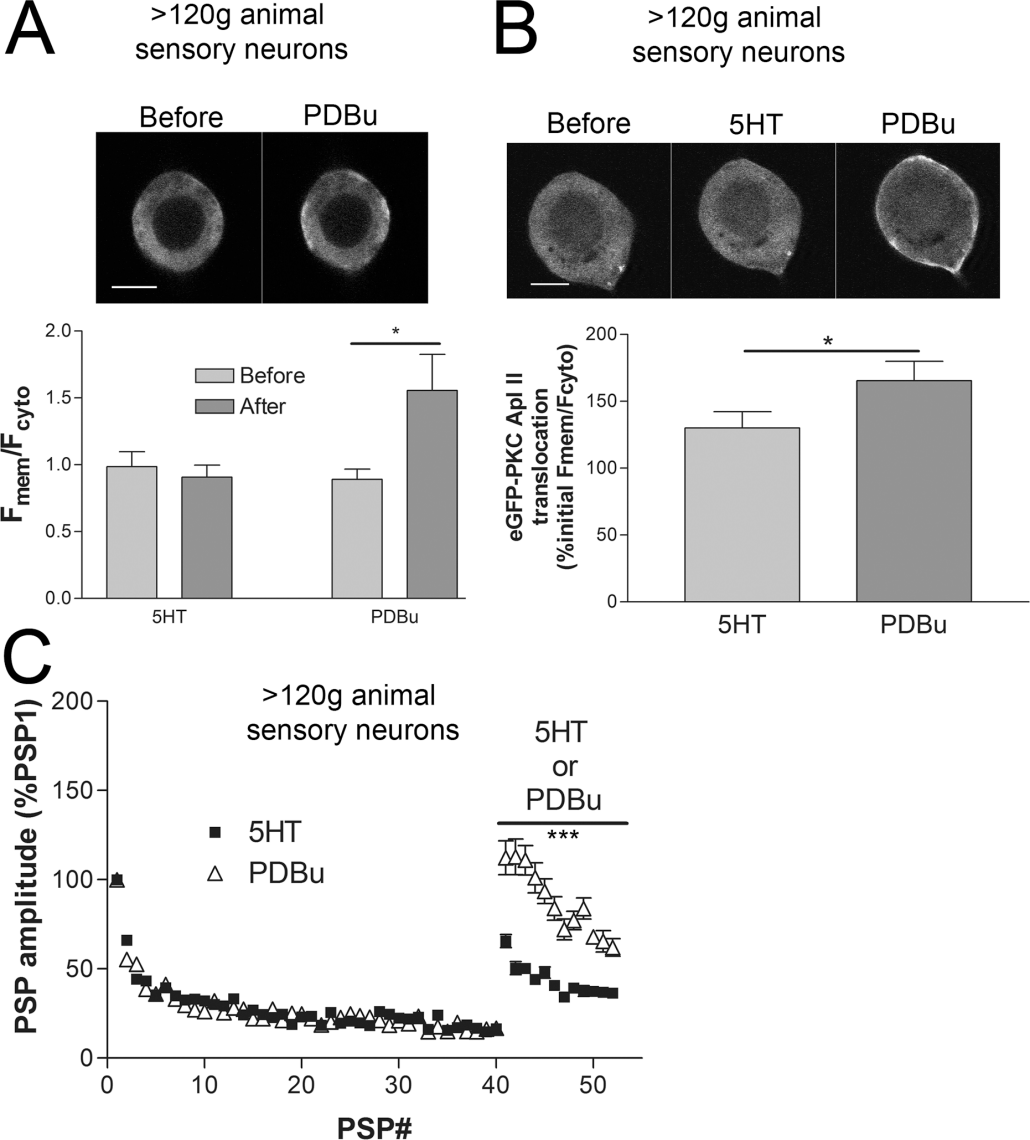
eGFP-PKC Apl II translocation and recovery from synaptic depression with PDBu from animals >120g. (A) Representative confocal images of a sensory neuron soma from an animal >120g expressing eGFP-PKC Apl II before and after application of 200nM PDBu. While 10μM 5HT did not result in an increase in the F_mem_/F_cyto_ ratio, 200nM PDBu significantly increased the F_mem_/F_cyto_ ratio at sensory neurons from >120g animals (comparing F_mem_/F_cyto_ before to after with a two-way ANOVA and Bonferroni post tests, 5HT P>0.05, PDBu P<0.05, n = 6 for each group). (B) In a second experiment, also with sensory neurons isolated from animals >120g, 5HT then PDBu were added sequentially with an image before, after 5min in 5HT, and after another 2min in 5HT + PDBu. Again, in this group of sensory neurons, 5HT had little effect on the eGFP-PKC Apl II translocation, however, the subsequent application of PDBu significantly increased translocation (P<0.05 comparing F_mem_/F_cyto_ with 5HT to PDBu with a paired t-test, data from 36 sensory neurons in n = 10 dishes). C) Homosynaptic depression of PSP amplitude and recovery from depression with 5HT or PDBu at synapses formed with sensory neurons from animals >120g. PDBu significantly increases facilitation following depression over 5HT (comparing the data sets with a two-way ANOVA and Bonferroni posttests, PSP1-PSP40 P>0.05 and PSP41-PSP52 P<0.001, n = 9 for 5HT and n = 7 for PDBu). Scale bars are 20**μ**m.

The previous experiment suggested that the impairment in PKC Apl II activation in the larger animals was upstream of PKC activation. If this is the case, then direct activation of eGFP-tagged PKC Apl II with a phorbol ester should rescue translocation of the kinase to the plasma membrane [16]. Cultures with neurons from animals >120g were treated with either 5HT or PDBu (200nM). Like the previous experiment, the sensory neurons isolated from animals >120g showed little to no translocation with 5HT. However, PDBu significantly increased the amount of eGFP-PKC Apl II translocation in the sensory neurons of these animals (Fig 5A). In a second experiment, we first added 5HT, measured the amount of translocation 5min later, then added PDBu and made a second measurement of eGFP-PKC Apl II translocation. As in the previous two experiments (Figs 4 and 5A), 5HT led to little translocation in sensory neurons isolated from the animals >120g, however, the subsequent application of PDBu significantly increased eGFP-PKC Apl II translocation (Fig 5B).

If PDBu can translocate PKC Apl II in the sensory neurons of the larger animals, where 5HT failed to activate the kinase, it should be able to rescue facilitation at depressed synaptic connections cultured from animals >120g. As described in Figs 1 and 3, 10μM 5HT led to incomplete recovery from synaptic depression when using sensory neurons from animals heavier than 120g (Fig 5C). However, direct activation of PKC Apl II with 200nM PDBu was able to recover PSP amplitude to original strength, and significantly more than with 5HT (Fig 5C). Since the independent observations of the eGFP-tagged kinase activation and the recovery from synaptic depression with 5HT and PDBu are consistent, we can conclude that the difference with the sensory neurons from the heavier animals lie in the signaling pathways upstream of PKC Apl II activation.

## Discussion

### Summary

Overall our data are consistent with previous behavioral findings that older/heavier animals show greatly reduced dishabituation, without a difference in habituation [9]. The 5HT-mediated recovery from synaptic depression is a key neuronal change underlying behavioral dishabituation of the defensive withdrawal reflexes [1,2]. When sensory neurons isolated from animals heavier than 120g are used to reconstruct synaptic connections in culture, the synapses show similar homosynaptic depression to low frequency stimulation but significantly less recovery from depression with 5HT (Figs 1 & 3). As the recovery from depression requires activation of the novel PKC Apl II in the presynaptic sensory neuron [8], impairment of its activation indicates a change upstream of kinase activation in the signal transduction pathway (Fig 4).

The *Aplysia* PKC Apl II, is a novel, calcium-independent PKC that is activated by diacylglycerol, phosphatidylserine, and phosphatidic acid binding to the kinase resulting in plasma membrane localization with activity [11,21]. Direct activation of PKC Apl II can be achieved with application of a phorbol ester [16]. Direct activation of the kinase with PDBu recovered translocation of eGFP-tagged PKC Apl II and facilitation following depression with sensory neurons from animals >120g suggesting that the difference in these neurons is upstream of PKC Apl II activation (Fig 5).

Since the activation of PKC Apl II by 5HT is complex, there are a large number of possible targets that could affect PKC translocation/activation. Inhibitors of phospholipase C (which is required for production of diacylglycerol), inhibitors of phospholipase D (which is required for production of phosphatidic acid) and inhibitors of tyrosine kinases are required for 5HT mediated translocation of PKC Apl II and the reversal of synaptic depression [11,18] and thus interruption of any of these pathways could explain the loss of PKC translocation in sensory neurons from animals >120g. Decrease in the level of the 5HT receptor coupled to PKC activation is another possible explanation. However, while activation of the 5HT_2Apl_ receptor is sufficient for translocation of PKC Apl II expressed in heterologous insect cells, antagonists of this receptor neither blocked the translocation of PKC Apl II in sensory neurons nor the reversal of synaptic depression [18], suggesting either a distinct unidentified receptor or a 5HT2 receptor complex with a pharmacological profile distinct from that present when the receptor is expressed in insect cells underlies physiological translocation in sensory neurons. This receptor complex and the associated signal transduction pathways are also highly regulated by other inputs: inhibitory transmitters block PKC Apl II translocation through reduction in phosphatidic acid production [13] and PKA activation downregulates the receptor complex [20]. Thus there are many possible loci for the animal to downregulate PKC Apl II translocation in sensory neurons.

The synaptic target of PKC Apl II that is responsible for the recovery from homosynaptic depression has also yet to be identified preventing monitoring of the phosphorylation and abundance of this target. Since PDBu largely rescues the reduction in the recovery from depression in the sensory neurons of the animals >120g, it is unlikely that the change in the larger animals is due to changes in the targets of PKC activation, as opposed to changes upstream of PKC activation. It is also unlikely that decreases in PKC Apl II itself could explain our results, as impairment in kinase activation was observed with expressed, exogenous PKC Apl II.

The possibility that our findings are the due to more damage to neurons isolated from large animals is unlikely since there were no differences in membrane excitability or in initial synaptic strength between small and large animals. While it is possible our results are specific to mariculture raised animals, a previous study on behavioral dishabituation found impairment in larger/older animals of both mariculture raised and field collected *Aplysia* [9], arguing against this possibility. There have not been published correlations between age/weight and the reversal of depression when the reversal of depression is measured in ganglia as opposed to dissociated cultures. It is conceivable that there are differences in the mechanistic details of the 5HT receptor complex between isolated neurons and neurons in the ganglia; the 5HT receptor antagonist spiperone has been reported to block reversal of depression in ganglia, but not in cultures [18,22], although this could also be a difference between the use of pleural and abdominal sensory neurons in the two experiments. It will be important in the future to determine the correlation between weight and reversal of depression when measurements are made in intact ganglia.

### Age-related impairment in memory or age/weight related change in behavior

*Aplysia californica* typically live about a year, initially as planktonic veliger then metamorphosing into a form resembling a small version of the adult when the proper food source is found [23]. The post-metamorphic juvenile will continue to grow over its life to weights often greater than a kilogram in the wild. So while the weight of an animal does relate to the age of the animal one cannot determine the absolute age of the animal from its weight as a result of a wide variety of factors including differences in growth rates between animals, nonlinear growth over the adult stages, and varying lengths of time in the planktonic and juvenile stages [10,23,24,25,26]. Highlighting this issue, in one of the cohorts used in Fig 3C of known age, animals ranged in weight from 83g to 460g. Thus, while it is more appropriate to refer to the age of an animal relative to the attainment of a particular developmental stage, this is not as useful for adults as there are only two stages (immature and sexually mature), and the transition between the two may be affected by animal weight [25]. When animals of known age were of similar size, no correlation between age and facilitation was found indicating that the change is not related to the absolute age of the animal. Had post-metamorphic age been known and compared to the recovery from depression (rather than post egg hatching age), a relationship would likely have been observed as post-metamorphic age would more strongly correlate with animal weight.

The relationship between the animal weight from which the sensory neurons are isolated and the amount of recovery from synaptic depression observed with 5HT shows that the reduction in facilitation starts in animals 100–200g. Animals in this weight range are becoming sexually mature (usually at around 160g in mariculture raised animals [25]). Thus, the reduction in the recovery from synaptic depression may have a potential reproductive function or support reproduction by reducing defensive mechanisms, promoting reproductive behaviors.

Another potential reason for the loss of dishabituation is a change in *Aplysia* predation with animal weight. The defensive withdrawal reflexes are important behaviors to protect the animal from injury and predation, however, as the weight of the animal increases the number of predators decrease with few predators of large adult *Aplysia californica* [27]. Two notable predators of *Aplysia californica*, the predatory slug Navanax and the Giant Green Sea anemone *Anthopleura xanthogrammica*, are only capable of predating on *Aplysia* less than about 75 grams and 100 grams respectively [28,29]. An analysis of *Aplysia californica* remains found in *Anthopleura x*. collected from a marine environment found an average weight of 52g and a maximum size of 100g [28] similar to the weight where we begin to see a reduction in the 5HT-mediated recovery from synaptic depression that underlies behavioral dishabituation.

Alternatively, the loss of dishabituation may represent an age-related impairment in synaptic plasticity. A variety of behavioral and neurophysiological changes have been reported in old *Aplysia*, usually in comparison to mature animals [9,10,30,31,32]. In these studies the impairments were in animals close to the end of their life and in comparison to sexually mature animals. Over the animal weight range that we find the most change in the recovery from synaptic depression (~100–200g), sensory neuron membrane excitability before and after 5HT was not significantly different. In a previous study of *Aplysia* pleural sensory neuron excitability, old *Aplysia* were found to have reduced membrane excitability compared to mature animals [32]. This difference is consistent with the change in the recovery from synaptic depression we report here being distinct from the changes previously reported and ascribed to animal senescence.

The loss of dishabituation with size of the animal is not the only developmental change in the plasticity of the reflex. Interestingly, very young animals (juvenile animals stage 9 to stage 11, less than 1g) are reported to not show facilitation following homosynaptic depression [33]. Dishabituation is seen in stage 11 and early stage 12 animals, but sensitization is not seen until later in stage 12 [34]. Unlike sensitization and dishabituation, habituation and the underlying homosynaptic depression of the sensory neuron synapses are observed at the earliest measureable stage (stage 9, less than 1cm juvenile animals) and persist in the oldest animals examined (>500g) [9,10,23,33]. Thus, similar to the gain of dishabituation seen in young animals, the loss of the reversal of depression in sensory neurons from ‘heavier’ animals is probably best described as a developmental change.
